# The enhancing impact of amino termini of hepatitis C virus core protein on activation of hepatic stellate cells 

**Published:** 2020

**Authors:** Khashayar Shahin, Seyed Younes Hosseini, Hoshang Jamali, Mohammad-Hossein Karimi, Negar Azarpira, Mastaneh Zeraatian

**Affiliations:** 1 *Gastroenterohepatology Research Center, Shiraz University of Medical Sciences, Shiraz* *, Iran*; 2 *Department of Bacteriology and Virology, * *Shiraz University of Medical Sciences* *, Shiraz, Iran.*; 3 *Department of Microbiology, Jahrom Branch, Islamic Azad University, Jahrom, Iran*; 4 *Transplant Research Center, Shiraz University of Medical Sciences, Shiraz, Iran*

**Keywords:** Hepatitis C virus, Hepatic stellate cell, core protein, liver fibrosis

## Abstract

**Aim:**

To investigate the potential effects of carboxyl and amino termini of HCV core protein on the HSCs activation.

**Background:**

The core protein is recognized as the most important fibrosis inducer of Hepatitis C virus (HCV). While the exogenous fibrotic effect of HCV core protein has been reported earlier, the endogenous effect and the role of two termini must still be investigated.

**Methods:**

Plasmids expressing full length, carboxyl-truncated (T1), or amino-truncated (T3) versions of the core were transfected into LX 2 cells. MTT assay was performed to evaluate the cytotoxicity of the endogenous expression of different regions of core protein on these cells. Afterwards, the total RNA was reversely transcribed and introduced into quantitative polymerase chain reaction (qPCR) to measure the expression level of collagen type I (COL1A1), α-smooth muscle actin (-SMA), tissue metalloproteinase inhibitor 1 (TIMP-1), and transforming growth factor-β1 (TGF-β1). In addition, TGF-β1 as a fibrotic factor, was also assessed in the supernatant of LX-2 cells using ELISA method.

**Results:**

The full and T1 versions of the core exhibited a measurable proliferative effect on LX 2 cells (P<0.05). Analysis of the gene expression was also showed that, in spite of amino-truncated version, these constructs represented a significant activation impact compared to the empty plasmid. Moreover, the result of TGF β assay was in agreement with the results of mRNA expression analysis.

**Conclusion:**

The endogenous expression of the full and carboxyl-truncated versions of the core exhibited a significant activator effect on HSCs. Therefore, it can be concluded that, amino domain of HCV core protein performs a stellate cell activation role.

## Introduction

 Since its discovery, Hepatitis C virus (HCV) remained as a unresolved health issue worldwide (1). The persistence moiety of HCV virus infection leads to liver fibrosis, cirrhosis, and hepatocellular carcinoma (HCC) (2). Liver fibrosis is characterized by unusual accumulation of extracellular matrix (ECM) substances promoted by hepatic stellate cells (HSCs) activation (-). The conversion of HSCs from quiescent phenotype into the activated form, which is a myofibroblast-like cell, occurs subsequent a damage or viral infections associated with more ECM production and inflammation. Over-expression of fibrosis-related mediators such as tissue inhibitors of metalloprotease (TIMPs), matrix metalloproteinase (MMPs), and collagen are results of HSCs activation (-). In general, in activated HSCs, the expressions of TIMPs are upregulated and lead to the inhibition of MMPs activity. Subsequently, matrix proteins such as collagen and α-smooth muscle actin proteins are extraordinarily collected in the extracellular space. In addition, synthesis and secretion of the fibrogenic cytokine and transforming growth factor β1 (TGF-β1) can initiate and intensify the fibrosis process (9, 10).

HCV genome encodes three structural (core, E1, E2) and at least six nonstructural proteins (-). Out of them, as the major determining factor of pathogenicity, core protein has attracted more attention in the fibrosis development. In fact, core as a capsid protein contains 2 main domains in amino and carboxyl terminal sides, which are responsible for host protein interaction and anchorage on membrane, respectively. Amino domain harbors a high basic sequence, which facilitates its interaction with host factors; and therefore, it could modulate the immune response, inhibit apoptosis, proceed cell transformation, and induce several biological pathways (14, 15). Despite performing intensive research on HCV pathogenesis; the exact role of proteins in fibrosis development has remained to be elucidated. It has been shown that the core protein is the most important fibrogenic molecule to induce the HSCs proliferation and activation, and it can occur through different pathways such as toll like receptor-2 (TLR-2) or obese receptor. The impacts of both endogenous and exogenous core on HSCs activation and consequent fibrogenic effect, have also been studied (3, 4, 7). However, considering the multifunctional activities of this protein, the roles of different domains of the core protein in activation of HSCs are not well defined yet. Previous research suggested that, for the establishment of liver fibrosis, the amino terminal of the core might be much more involved in critical interactions with a range of host proteins than the carboxyl terminal region (16).

The main aim of this study was to assess the potential effects of carboxyl or amino terminals of core protein on HSCs activation. 

## Methods

**Cell culture**

The immortalized human HSC LX-2 cell line (courtesy of Dr SL Friedman, Mount Sinai School of Medicine, New York, NY, USA) (17) was cultured in low glucose Dulbecco’s modified Eagle’s Medium (DMEM, Gibco USA) supplemented with 4% fetal bovine serum (FBS, Sigma, St. Louis, USA), 100 U/ml penicillin-streptomycin (Gibco USA) and 2mM L-glutamin, and then incubated at 37 °C in 5% CO2 air humidified atmosphere.

**Plasmid constructs**

The expression plasmids (courtesy of Dr. Gloria González-Aseguinolaza, Gene therapy and Hepatology department, CIMA Research Centre, Pamplona, Spain) were originally modified from an AAV shuttle vector (Clontech Inc.). Plasmids AAV-EF-full-core, AAV-EF-T1, and AAV-EF-T3 are producing full length core (aa 1-190), carboxyl-truncated core (aa 1-135), and amino-truncated core (aa 50-190), respectively. Plasmid AAV-EF carries no part of the core protein sequence (it was used as the negative control in all experiments. The expression of all plasmids had been evaluated using immunohistochemistry or reverse transcriptase quantitative real-time PCR (RT qPCR) assay (unpublished data).

**Transfection of LX-2**

A total number of 3 × 10^5 ^cells/well was plated in 24-well culture plates a day before transfection. The plates were incubated with fresh complete medium one hour before performing the plasmid transfection. The transfection was performed using X-tremeGENE HP^TM^ DNA transfection reagent (Roche Inc, Germany) in a serum free DMEM medium in terms of the manufacturer’s instructions. After 6 hours of incubation, complete fresh medium was replaced in each well, and was then incubated for further 24 h at 37 °C in a humidified atmosphere. A set of wells (3 biological replicate) containing the culture media supplemented with previously verified dose of human leptin (75 ng/ml, Sigma Aldrich; Merck KGaA, Darmstadt, Germany) and a pro fibrotic hormone were used as the positive control group. Another set, which contained transfected cell by AAV-EF plasmid, was also used as the negative control group.

**RNA extraction and reverse transcription**

After the transfection, the cells were harvested by centrifugation and the total RNA was extracted using Trizol reagent (Invitrogen, Thermo Fisher Scientific, Inc. USA) in terms of the manufacturer’s instructions. The extracted RNA was eluted in 50µl of RNAse-free water and stored at -80 °C until further usage. To synthesize cDNA, the RNA was treated with RQ1 DNase (Promega Corporation, Madison, WI, USA) to remove the residual DNA contamination. Afterward, the cDNA was synthesized using RocketScript RT premix kit (Bioneer Corporation, Daejon, S. Korea) in terms of the manufacturer’s protocol. Briefly, 1µg of the DNase-treated RNA was added into the reaction tubes containing a mixture of reverse primers for TIMP-1, COL1A1, α-SMA, TGF-β1, and GAPDH (18). The tubes were heated to 60 ºC for 1 min followed by 60 min incubation at 45 ºC for reverse transcribing. The produced cDNA was stored at 20 ºC until use in real-time PCR reactions.

**Real-Time quantitative polymerase chain reactions**

The mRNA levels of COL1A1, TIMP1, TGF-β1, and α-SMA were analyzed using SYBR green I master mix (Bioneer Corporation, Daejon, Korea) by IQ5 Real-time PCR system (Biorad Inc., USA) and using earlier reported primer pairs (10). Glyceraldehyde-3-phosphate dehydrogenase (GAPDH) was used as the housekeeping gene. Positive and negative controls were also included in all steps to ensure the accuracy of the experiments. The amplification reaction was 40 cycles of 10 seconds at 94 °C for denaturation and 20 seconds at 58 ºC for annealing and extension steps. Moreover, for each qPCR run, a melting curve analysis and a gel electrophoresis were performed to ensure the accuracy of the method. Amplification signals for different mRNAs were normalized using the obtained signals for the housekeeping gene, GAPDH. Also, all reactions were performed in triplicate and data were analyzed using the 2^−ΔΔCt ^ method as described elsewhere (19).

**Cell viability assay**

MTT assay was used to assess the effects of different expressing plasmids on the activated-LX-2 cell proliferation. A 96-well microtitre plate was seeded with 7.5×10^3^ LX-2 cells/well. After reaching a confluence of 90%, the cells were transfected with plasmids. After 24 hours incubation at 37 ºC, the supernatant of each well was replaced with a mixture of 90 µl fresh medium and 10µl of a 5 mg/mL MTT solution [3-(4, 5-dimethylthiazole-2-yl)-2, 5-diphenyl tetrazolium bromide] (Sigma Aldrich; Merck KGaA). Then, 100µl DMSO (Dimethylsulfoxide) (CinnaGen, Inc., Tehran, Iran) was added to each well, and the plate was re-incubated on a horizontal rotator to dissolve formazan crystals. Finally, microplate ELISA reader (BioTek Elx 808; BioTek Instruments, Inc., Winooski, VT, USA) was used to read the absorbance at two wavelengths of 570 and 630 nm  (20). 

**TGF-β1** **measurement by ELISA assay**

 By passing 24 hours from the transfection, the TGF-β1 cytokine level was measured in the culture supernatant using a Human-Mouse TGF-β1 ELISA kit (eBioscience, Inc.; Thermo Fisher Scientific, Inc.) in terms of the manufacturer's instruction    (21). In brief, to activate TGF-β1 protein, the supernatant of each sample was acidified for 20 minutes at room temperature, and was then captured and detected by adding antibodies. The absorbance was measured at 450 nm using ELISA reader (BioTek Instruments, Inc.; Elx 808), and the data were calculated against the standard curve and adjusted to pg/ml of culture medium. All experiments were performed in triplicate.

**Statistical analysis**

Data preparation and statistical analysis were performed using Microsoft Excel 2013 and GraphPad Prism (V 5.0, GraphPad, USA). One-way ANOVA with proper correction (Tukey test) was used for comparing the data and a P≤0.05 was considered as significant. 

## Results

**C- and N-Terminus of core protein induced different expression profiles of fibrogenic genes**

**Figure 1:**
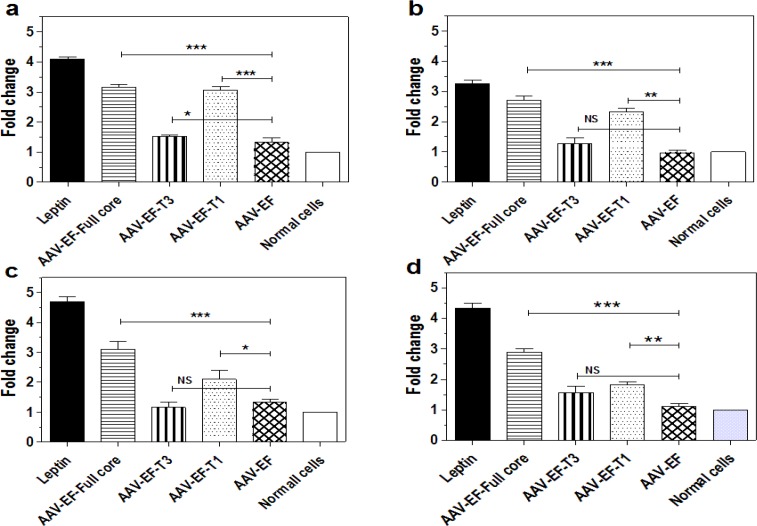
The Effects of different parts of core protein on the genes expression patterns of LX-2 cells. (a) COL1A1. (b) TIMP1. (c) TGF-β1. (d) α-SMA. The mRNA levels of these genes were measured by qPCR subsequent the transfection by AAV-EF-Full, AAV-EF-T1, and AAV-EF-T3 plasmids. AAV-EF transfected cells and leptin-treated cells were enrolled as negative and positive controls, respectively. Bars are representatives of the standard deviation of three independent experiments. * P < 0.05, ** P < 0.01, and *** P < 0.001 indicated the significant comparison with negative control group (AAV-EF)

**Figure 2:**
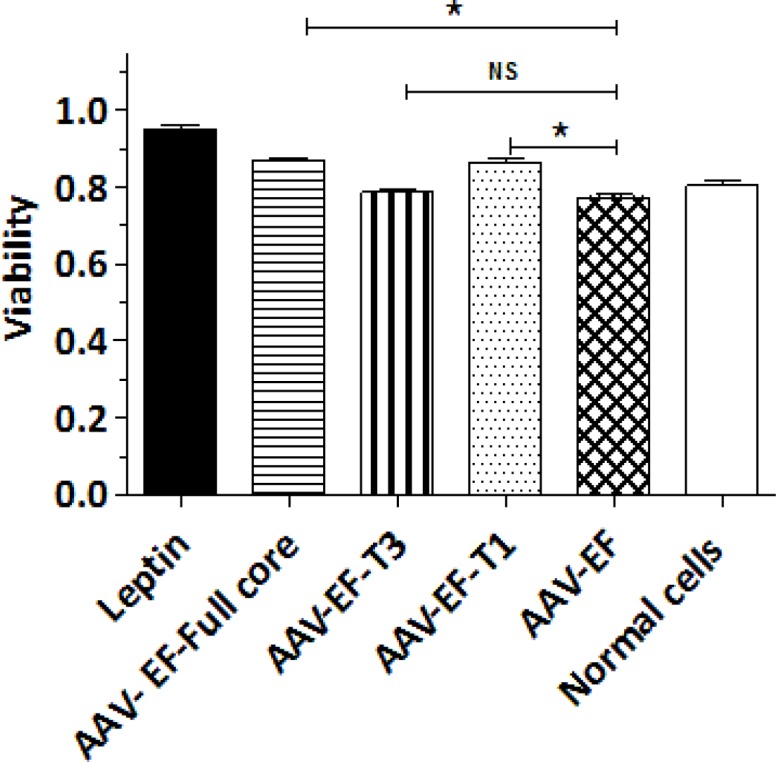
LX-2 cell proliferation assay after transfection with AAV-EF-core full, AAV-EF-T1, and AAV-EF-T3. * P < 0.05 indicated the significant level in comparison with the negative control group (AAV-EF)

To understand the effects of different regions of HCV core protein on the expression profile of fibrotic genes, the expression levels of COL1A1, TIMP1, TGF-β1, and α-SMA were measured by qPCR. In the transfected cells, complete, carboxyl-truncated (AAV-EF-T1), and amino truncated (AAV-EF-T3) HCV core proteins increased the expression level of COL1A1 by 3.16, 3.06, and 1.56 times, respectively (Figure 1-a). The expression level of TIMP-1 up-regulated by 2.7, 2.3, and 1.26 times (Figure 2-b), for TGF-β1 it increased by 3.1, 2.1, and 1.16 folds (Figure 1-c), and for α-SMA, as the main marker of fibrosis, it increased by 2.9, 1.83, and 1.53 folds, respectively (Figure 1-d). In the negative control, no significant up-regulation was observed in the cells transfected with empty AAV-EF plasmid compared to normal cells.

**The effect of different parts of HCV core protein on LX-2 viability**

To investigate the endogenous expression effects of different parts of HCV core protein on LX-2 proliferation; the cell viability (as a feature of liver under fibrosis progression) was analyzed after performing the transfection. The MTT results clearly demonstrated that, the viability of LX-2 cells was improved by transfection of AAV-EF-Full core as well as AAV-EF-T1 (P< 0.05) (Figure 2). However, there was no significant increase in the cells proliferation, which were transfected with AAV-EF-T3, and this suggests that, the cell proliferation was not endogenously affected by the amino truncated HCV core protein (Figure 2).

**Effect of different parts of HCV core protein on TGF-β1 production**

**Figure 3:**
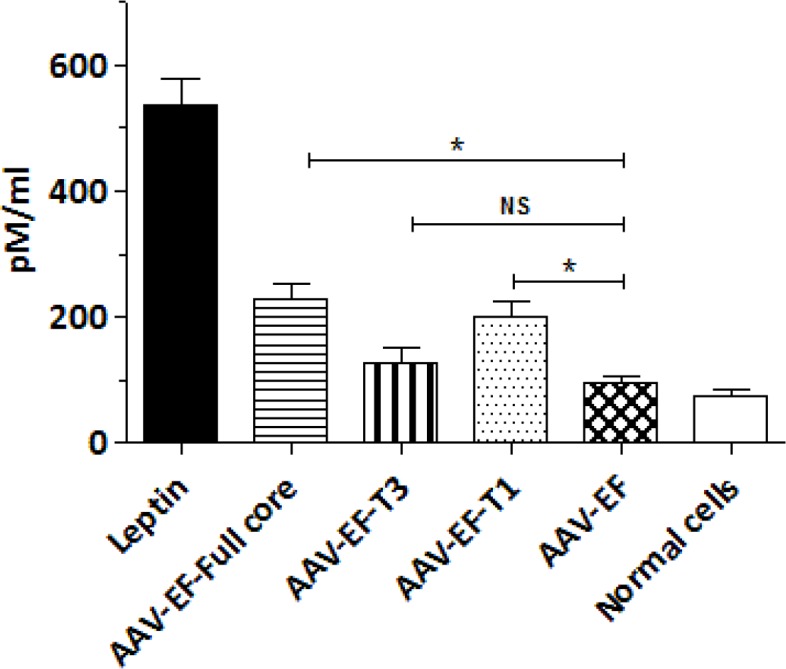
Effect of different parts of HCV core protein on TGF-β1 production. AAV-EF-Full core and AAV-EF-T1 induced a significant increase in TGF-β1 production. Each bar is a representative of the mean ± standard deviation of at least three measurements. Leptin treated and AAV-EF transfected cells were used as positive and negative controls, respectively. *P < 0.05 showed significant levels compared to the control group (AAV-EF)

The level of TGF-β1, as a pro-fibrotic cytokine, was measured in the cell culture supernatants after being transfected with the aforementioned plasmids using ELISA. Production and secretion of TGF-β1 by LX-2 cells were significantly affected when transfected with AAV-EF-Full (229 pg/ml) or AAV-EF-T1 (199 pg/ml) (P< 0.05). In contrast, AAV-EF-T3 transfected LX-2 cells showed no significant changes in TGF-β1 production compared to the negative control cells (AAV-EF) (Fig. 3).

## Discussion

Although chronic infection with HCV and hepatitis B virus (HBV) can cause liver fibrosis, the fundamental mechanisms are not well understood (22). It is suggested that, this process is mainly related to direct activation of HSCs by some viral proteins such as NS3, NS5, and core proteins of HCV and HBX from HBV virus (3). Considering the significant role of HSCs in the fibrosis process, understanding the interaction of HCV proteins with HSCs might be useful in explaining the fibrosis mechanism by this virus (7). In the case of HBV virus, it has been recently demonstrated that, while HBX expression induces activation of the LX-2 cell, pre-core protein induces no LX-2 cells activation (23). 

HCV proteins have been considered to investigate their activation properties on stellate cells. More recently, Khanizaedeh et al. reported that, endogenous expression of NS3 protein in LX-2 cell has an activating effect (24). Likewise, a fibrotic role of NS3 protein on HSCs cell was shown in other in vitro experiments (3). However, core protein takes more consideration as a critical molecule with a fibrogenic role (4, 7). It has been shown that, the treatment of HSCs with the core protein can induce the fibrotic phenotype; however, the role of amino or carboxyl termini of protein in this process has yet to be explained. 

The present study aimed to determine the fibrogenic effects of intracellular expression of core protein on LX-2 activation, and to discriminate between the effects of amino or carboxyl termini on this process. Up to the best of our knowledge, this is the first study looking into the fibrotic role of different regions of the HCV core gene on the HSCs.

The LX-2 cell is an immortalized stellate cell line appropriate for the assessment of stellate cell behavior during the normal activity or fibrogenesis (17). Previously, this cell line was used to investigate the fibrotic effect of HCV core protein where the core protein showed a profound effect on HSC activation (7). The interaction of HCV core and HSCs, and the endogenous or exogenous fibrotic effects of the core protein on the primary human HSCs or LX-2 were also investigated earlier (3, 17). In agreement with these studies, which have shown that core protein acts as a fibrotic molecule since it increases the expression level of collagen, TIMP-1, MMPs, TGF-b, and other markers of HSCs activation; our study demonstrated a similar effect in which gene expression levels of fibrotic genes in LX-2 cell transfected with core expressing plasmid were significantly increased. Accordingly, Bataller et al. reported a similar effect after transduction of LX-2 with an adenovector expressing the core protein (3). Moreover, Coenen et al. reported a similar trend for COL1A1 expression level in the LX-2 cell line treated with exogenous core protein (7). 

The core gene encodes capsid protein with a unique structure and a multifunctional property (16). The amino terminal region (Domain I) is enriched with basic amino acids, which can interact with both viral and host proteins; thus, engaging the Core in several regulatory roles on apoptosis, cell cycle progression, un-controlled division , and cell inflammation (-). Carboxyl terminal region (Domain 2) interacts with lipid droplets and membranes to make replication complex, and is also crucial for virus morphogenesis (29). 

In the current study, carboxyl and amino-truncated forms of the core gene were investigated to understand the role of each region during the fibrosis process. Interestingly, the noticeable changes in the fibrotic gene expression level following transfection with AAV-EF-T1 (carboxyl-truncated) imply a lower effect for carboxyl region of the core in the activation of LX-2 cell line. This activation pattern was similar to that of the full core expression in a sense that, the endogenous expression of carboxyl-truncated core significantly increased the mRNA level of all of the investigated fibrotic genes. In other words, it supports the role of amino terminal in fibrogenesis process. In contrast, using the transfected cells with AAV-EF-T3 (amino-truncated core) showed no significant change in the expression level of such genes; in other words, carboxylic terminal played no significant role in fibrogenesis. 

The viability assay demonstrated that the endogenous expression of the full core protein in LX-2 cells resulted in a significant increase in the cell proliferation. Butler et al. reported an increased cell proliferation due to HCV core; however, it was not for NS3-NS4 protein (3). On the other hand, one study demonstrated that the core protein had no effect when added into culture media of HSC cell (7). Our study also showed that, similar to the effects of the full protein, the carboxyl-truncated form enhanced the cell proliferation, while the amino-truncated core protein had no significant effects. This finding emphasized again on the importance of the amino terminus of core protein in the HSC activation.

Furthermore, TGF-β1 production was endogenously stimulated by the full and carboxyl-truncated HCV core protein, which is in agreement with other studies where higher TGF-β1 was produced in the transfected cells by the core or NS3 proteins, respectively (3, 30). Hence, we showed that, the amount of TGF-β1 secretion by transfected cells with AAV-EF-T3 was not statistically significant. Also, the regulatory role of core protein domains has been investigated in previous studies (-). The amino terminal domain of the HCV core protein has been assigned to be responsible for the majority of the regulatory roles (28).

In conclusion, this study demonstrated that, while all core constructs induced a degree of HSCs activation, this potency was significantly ablated for amino-truncated core protein compared to full core. In other words, the amino terminal region of core protein, which is harboring the basic sequence, is important in the activation of HSCs that had not been mentioned earlier. It is possible that, core interaction through domain 1 with other host factors trigger the pathways involved in HSCs activation. However, conducting further studies are required to address the limitations of the current study including absence of protein assay in expression analysis as well as animal study to establish a stronger scientific ground for such a role.
